# Notes and News

**Published:** 1893-09-23

**Authors:** 


### Notes and News.

Collected and Communicated.

The Clent branch of the Order of Foresters is setting a good example to kindred societies by the erection of a new convalescent home for its members A small institution of this kind has been in existence for some time, but the new building, of which the foundation-stone was laid a few weeks since, will be on a larger scale, and provide accommodation for thirty patients. The Committee are appealing for help from the public, that the home may be opened free from debt. The members themselves are doing their utmost towards this end, and have already subscribed £1,000 towards the £2,500 required. Such efforts on their part deserve every encouragement, and we hope the necessary funds may soon be forthcoming.

Under the terms of the "William F. Jenks Memorial Trust" the third triennial prize of five hundred dollars will be awarded by the College of Physicians of Philadelphia next year to the author of the best essay on "Infant Mortality during Labour, and its Prevention." The conditions laid down by the founder of the prize are that it "must always be for some subject connected with obstetrics, or the diseases of women, or the diseases of children." The publication of the successful paper rests in the discretion of the trustees, and if not published it remains the property of the college. The prize is open for competition to the whole world but the essay, which must be the production of one person, must be written in the English language or accompanied by an English translation, and must be sent to the College of Physicians of Philadelphia, Pennsylvania, U.S.A., before January 1st, 1895, addressed to Horace Y. Evans, M.D., Chairman of the Prize Committee. "Each essay must be typewritten, distinguished by a motto, and accompanied by a sealed envelope bearing the same motto and containing the name and address of the writer." Only that one belonging to the successful essay will be opened. The committee have the right to make no award should none of the papers be considered worthy of the prize.

The Queen has sent presents of wine for the use of the patients in the following hospitals: The Seaman's Hospital, Greenwich, the National Hospital, Queen Square, the London, the Cancer, the Royal Chest, the Samaritan Free, and the British Home for Incurables.

A large bazaar was opened last week at Blackpool by Sir Mathew White Ridley, in aid of the funds for the new hospital in that town now in course of erection.

Hospital literature of various kinds steadily increases in volume. We have to announce the publication of the "Leeds Hospital Magazine," under the direction of the Committee of the Leeds Working People's Fund, the first number of which will be issued on October 1st next. It is published at one halfpenny, will contain sixteen pages 4to., and will be supplied for twelve months, postage free, for one shilling, payable in advance, to the Editors, care of City Printing Works, Cookridge Street, Leeds.

A young man recently found himself out of work and out of money at Cardiff, and therefore, like many another prodigal, he began to think of his home, a humble enough place, in a Hampshire village. He turned his back on the town of his adoption, and set off "on the tramp," and during the days which followed he lived only on apples. "It was such a good year for them," he gratefully acknowledged, and he received from many a cottager all he could eat or carry away. Except that he felt "a bit low" by the time he reached the parental roof, his physical condition was undoubtedly excellent, and might be taken as one amongst many examples that sound, fresh fruit is harmless. Of course, a diet composed exclusively of apples might eventually prove both monotonous and dangerous, but it is free from the perils which attend the terrible masses of rottenness which attract the London poor, tempted by apparent cheapness, to invest in penn'orths of plums, and such like, which might justly be denominated as the true "cholera mixture."

Anyone wanting help instinctively asks it of a busy person, not because the latter is already fully occupied but from him (or her) being probably most capable of giving the aid or assistance required. The inevitable request "to spare a little time" invariably is acceded to by the promise "to make time," to do what is wanted. There is something characteristic in the latter expression, and a person who uses it seldom fails to carry out his undertaking. With regard to "spare" time, those who have most generally use it least. When fashionable people dabble in what they call "charity" they are apt to consider the work taken up under this all-embracing word as an adjunct to their other occupations, and when these become too pressing the district or hospital visits are dropped because the ladies cannot "spare" time for them, and they have not the busy woman's faculty for "making" a little leisure. "When they take up work they ought to make it their first duty, not the last," said the head of a band of voluntary helpers the other day. Surely work undertaken nominally for "love" should be at least as conscientious and systematic as any that is done merely for "money."

